# Application of Genomic Tools in Plant Breeding

**DOI:** 10.2174/138920212800543084

**Published:** 2012-05

**Authors:** A.M. Pérez-de-Castro, S. Vilanova, J. Cañizares, L. Pascual, J.M. Blanca, M.J. Díez, J. Prohens, B. Picó

**Affiliations:** Instituto de Conservación y Mejora de la Agrodiversidad Valenciana, Universitat Politècnica de València, Camino de Vera 14, 46022 Valencia, Spain

**Keywords:** Bioinformatics, complex traits, genetic maps, marker assisted selection, molecular markers, next-generation-sequencing, quantitative trait loci.

## Abstract

Plant breeding has been very successful in developing improved varieties using conventional tools and methodologies. Nowadays, the availability of genomic tools and resources is leading to a new revolution of plant breeding, as they facilitate the study of the genotype and its relationship with the phenotype, in particular for complex traits. Next Generation Sequencing (NGS) technologies are allowing the mass sequencing of genomes and transcriptomes, which is producing a vast array of genomic information. The analysis of NGS data by means of bioinformatics developments allows discovering new genes and regulatory sequences and their positions, and makes available large collections of molecular markers. Genome-wide expression studies provide breeders with an understanding of the molecular basis of complex traits. Genomic approaches include TILLING and EcoTILLING, which make possible to screen mutant and germplasm collections for allelic variants in target genes. Re-sequencing of genomes is very useful for the genome-wide discovery of markers amenable for high-throughput genotyping platforms, like SSRs and SNPs, or the construction of high density genetic maps. All these tools and resources facilitate studying the genetic diversity, which is important for germplasm management, enhancement and use. Also, they allow the identification of markers linked to genes and QTLs, using a diversity of techniques like bulked segregant analysis (BSA), fine genetic mapping, or association mapping. These new markers are used for marker assisted selection, including marker assisted backcross selection, ‘breeding by design’, or new strategies, like genomic selection. In conclusion, advances in genomics are providing breeders with new tools and methodologies that allow a great leap forward in plant breeding, including the ‘superdomestication’ of crops and the genetic dissection and breeding for complex traits.

## INTRODUCTION

Ever since the beginnings of the domestication of plants, some 10,000 years ago, plant breeding has been extremely successful in developing crops and varieties that have contributed to the development of modern societies, and have successively beaten (neo-)Malthusian predictions [1]. Application of conventional pre-genomics scientific breeding methodologies has led to the development of modern cultivars, which have contributed to the dramatic improvement of yield of most major crops since the middle of the 20^th^ century. The success of plant breeding in the last century has relied in the utilization of natural and mutant induced genetic variation and in the efficient selection, by using suitable breeding methods, of the favorable genetic combinations. In this respect, the evaluation and identification of genetic variants of interest as well as the selection methodologies used have largely been based in the phenotypic evaluation. 

Nowadays, genomics provides breeders with a new set of tools and techniques that allow the study of the whole genome, and which represents a paradigm shift, by facilitating the direct study of the genotype and its relationship with the phenotype [2]. While classical genetics revolutionized plant breeding at the beginning of the 20^th^ century, genomics is leading to a new revolution in plant breeding at the beginning of the 21^th^ century. 

The field of genomics and its application to plant breeding are developing very quickly. The combination of conventional breeding techniques with genomic tools and approaches is leading to a new genomics-based plant breeding. In this new plant breeding context, genomics will be essential to develop more efficient plant cultivars, which are necessary, according to FAO, for the new 'greener revolution' needed to feed the world’s growing population while preserving natural resources. 

One of the main pillars of genomic breeding is the development of high-throughput DNA sequencing technologies, collectively known as next generation sequencing (NGS) methods. These and other technical revolutions provide genome-wide molecular tools for breeders (large collections of markers, high-throughput genotyping strategies, high density genetic maps, new experimental populations, etc.) that can be incorporated into existing breeding methods [2-5]. Recent advances in genomics are producing new plant breeding methodologies, improving and accelerating the breeding process in many ways (e.g., association mapping, marker assisted selection, ‘breeding by design’, gene pyramiding, genomic selection, etc.) [5-7]. 

Genomics approaches are particularly useful when dealing with complex traits, as these traits usually have a multi-genic nature and an important environmental influence. Thanks to these technological improvements it is now feasible for a small laboratory to generate in a short time span (e.g., several months) enough molecular data to obtain a set of mapped quantitative trait loci (QTLs), even in a species lacking any previous genomic information [8]. Genomic tools are thus facilitating the detection of QTLs and the identification of existing favorable alleles of small effect, which have frequently remained unnoticed and have not been included in the gene pool used for breeding [9, 10]. 

In this review, we present and discuss the most relevant advances in the development and application of genomic tools for plant breeding, in particular for complex traits. Firstly, we introduce the most relevant genomic tools and secondly we provide examples of the application of these tools to plant breeding. The objective is to provide modern breeders with an updated synthetic view of how genomics can effectively improve the efficiency of breeding programs.

## GENOMIC TOOLS AND RESOURCES FOR PLANT BREEDING

### Genome and Transcriptome Sequencing

The availability of the whole genome sequence of a crop is of great utility for plant breeding, but until recently it has been an unachievable goal for most crops. This privilege was restricted to a reduced number of model species with small genomes and to projects with an important investment in time and resources, but now has extended to an increasing number of crops. However, it is also true that for important cultivated species with large and complex genomes such as wheat, sugarcane, or coffee, the sequencing of the whole genome is very challenging and may take several years before a draft is completed. Given the high cost of whole genome sequencing, transcriptome sequencing has been a cheaper alternative. The cDNA sequences (expressed sequence tags, ESTs) provide relevant information about the genes expressed in a certain tissue or organ, at a given stage of development and under particular environmental conditions. ESTs sequencing projects do not provide information about non-coding sequences and, even using diverse libraries, it is difficult to identify all genes and transcripts variants. Despite these limitations, ESTs collections have been very useful for breeders. 

The Sanger technology has been the predominant sequencing method for the past thirty years. It has been used to sequence several genomes as well as many transcriptomes. The first international collaborative project resulted in the whole genome sequence of the model plant *Arabidopsis thaliana* [11]. Since then, reference genomes of selected genotypes were completed in a limited number of crops such as rice [12], maize [13], sorghum [14], populous [15], grapevine [16], papaya [17], or soybean [18]. The transcriptomes of most major crops, to a greater or lesser extent, were also sequenced. A global view of the genomes and transcriptomes sequenced can be obtained from the Gene Index Project (http://compbio.dfci.harvard.edu/tgi/plant.html) or in the NCBI Unigene database (http://www.ncbi.nlm.nih.gov/unigene). 

The field of genomics has changed with the arrival of NGS technologies [19]. These new technologies have reduced the cost of sequencing by more than one thousand times compared to Sanger technology, by avoiding time-consuming and tedious traditional cloning steps and making possible to perform millions of sequencing reactions in parallel (Table **1**). Among the “second generation” technologies, the 454 (Roche, http://www.454.com) and Illumina (Illumina, http://www.illumina.com) platforms are already profusely used to sequence crop species. Others, like Solid (Applied Biosystems, http://www.appliedbiosystems.com/technologies), have been less exploited in plants. By using these NGS technologies, the sequencing capacity of laboratories is continuously increasing. For instance, one High-Seq 2000 Illumina Sequencer is able to generate 55 Gb per day, which is roughly eighteen times the size of the human genome. Moreover, new, “third generation” platforms are being developed and incorporated to sequencing projects, such as PacBio RS (Pacific Biosciences, http://www.pacificbiosciences.com), Helicos (Helicos, http://www.helicosbio.com), or Ion Torrent (Life Technologies, http://www.iontorrent.com). The sequence obtained by NGS are generally deposited in the NCBI Sequence Read Archive (http://www.ncbi.nlm.nih.gov/unigene).

Nowadays, it is feasible to sequence most crop genomes (excluding those with a very large and complex genome) with a relatively modest budget, by combining Sanger with NGS technologies. Sequencing projects for 135 plant genomes, most of them corresponding to cultivated species or wild relatives, have been completely sequenced (3), are being assembled (27) or are in progress (105), as reported at the NCBI database (http://www.ncbi.nlm.nih.gov/genomes/leuks.cgi). Other databases including plant genome sequences are Plant GDB (http://www.plantgdb.org) and Phytozome (http://www.phytozome.net). A fully sequenced and well annotated genome provides useful tools for the breeders, as it allows the discovery of genes, determining their position and function, as well as the development of large marker collections and high resolution maps. In the cases where only a draft sequence is available, its usefulness depends on the quality of the assembly. For instance on many occasions thousands of scaffolds are obtained but they are not anchored to the genetic map, which difficults the use of the information. Many transcriptomes have also been sequenced, a number of them in species for which no previous sequence information was available. Sweet potato [20], squash [21], pigeonpea [22], or buckwheat [23] represent some examples published in the last months. These assays are showing the great complexity of plant transcriptomes, allowing the identification of rare transcript variants that are being used to improve gene annotation and our knowledge of gene function and regulation. 

### Bioinformatics

NGS technologies are facilitating sequencing projects, but have brought new challenges, as millions of short DNA reads have to be analysed and assembled [19]. Also, genetic maps, genotypes, or expression information at a genomic scale have to be processed in order to obtain the relevant biological information. Therefore, it is necessary to develop new bioinformatics tools (algorithms and software), which allow the analyses of huge amounts of genome-wide data, but it is also necessary to change the approaches used to understand complex biological traits [25, 26]. 

The field of sequence analysis has a long tradition and has enabled the assembly of many genome sequences obtained by Sanger sequencing. Nowadays, the huge amount of sequence reads generated by NGS and the low quality of individual reads requires new software tools and algorithms that allow dealing with these data in an efficient way [27]. We consider this to be a limiting factor for this kind of analyses right now. Although in the last years great advancements in the sequence processing algorithms have been achieved, the software currently available requires improvements in many cases. 

Two of the most common analyses carried out on these NGS reads are genome assembly and annotation and mapping. Genome assembly is a complex task requiring powerful computers and skilled bioinformaticians [25]. In particular, the RAM memory requirements of the computers used to assemble eukariotic genomes could hinder the application of this technique by small laboratories. Some of the most commonly used assemblers are Roche's 454 Gsassembler, Celera Assembler, and Mira. Once a reference genome is available in the species it is common to study its variation [19]. To do this, a mapper software is commonly used instead of an assembler software. A mapper tries to align every read against the reference genome. This process is much simpler and faster than the assembly. In this case the computer requirements are usually less demanding and the limiting factor could be the storage capacity. Some commonly used mappers are Bowtie, BWA, and TopHat. Once the reads are aligned, single nucleotide polymorphism (SNPs) can be detected by using the SAMtools or the GigaBayes SNP callers [28].

The open source software mainly used by the bioinformaticians is cumbersome for users not well versed in the Unix command line operating system. Some commercial proprietary solutions easier to use have been developed (e.g., LaserGene or CLC Genomics Workbench), but they have not been widely embraced by the breeders. Galaxy is an open source project devoted to create an easy to use web interface to the open source CLI based applications used in this area.

An important amount of work has been devoted in this field to the creation of standard and open file formats capable of storing information regarding sequence alignment and modelling (SAM) [24], SNP calls using variant call format (VCF; http://1000genomes.org/wiki/doku.php?id=1000_genomes: analysis:vcf4.0), genomic regions with browser extensible data (BED, http://genome.ucsc.edu/FAQ/FAQformat# format1) and genomic annotations using the general feature format (GFF; http://www.sequenceontology.org/resources/gff3.html]). These open and standard formats allow the interoperability of the different software tools that are being actively developed and used. In addition, the computer requirements might be strong as some analyses require a large amount of RAM memory or storage capability.

The algorithms and methods used to store and process raw genomic data generated by the different technological platforms will depend on the type of data being processed and on the result expected. In any case, once the relevant information is obtained by the bioinformaticians, results have to be made available to the breeders by using an interface as easy and friendly as possible [25]. To provide access to this information, the generation of an easily browseable web site is a common and usually successful approach. Several general purpose web databases exist to make the relevant biological information available to the researchers and breeders (Table **2**), like GenBank (http://www.ncbi.nlm.nih.gov/genbank/), EBML (http://www.ebi.ac.uk/embl/), DDBJ (http://www.ddbj.nig.ac.jp/) and Swiss-prot (http://expasy.org/sprot/). These latter databases are devoted to store information about any species, but several other more specific databases focused on species of interest to the breeders also exist, like the Sgn (http://solgenomics.net/), Phytozome (http://www.phytozome.net/), Gramene (http://www.gramene.org/) or CropNet (http://ukcrop.net/), which hold information that could have more specific use for breeding programs.

### Expression Studies, from Microarrays to RNA-seq

New genomic tools are also of interest to expand and accelerate gene expression studies. The analysis of gene expression has provided a rich source of biological information, which allows breeders to understand the molecular basis of complex plant processes, leading to the identification of new targets for manipulating these processes. 

Gene expression studies were at first based on the classical Northern blot method that only allowed the quantification of tens of genes simultaneously. The QRT-PCR is a more affordable and quantitative technique; but the number of genes analyzed by experiment is also limited [29]. Other approaches allowing the study of thousands of genes were differential display and cDNA amplified fragment length polymorphisms (cDNA-AFLPs) [30]. However, these methods are not really quantitative and are limited by the ability of the developed libraries to capture low-abundance transcripts. Other methods that overcome part of these problems are the serial analysis of gene expression (SAGE) [31] and massively parallel signature sequencing (MPSS) [32]. Nevertheless, the most employed methods at present to analyze transcript profiling are the hybridization-based platforms or microarrays [33]. Expression arrays have several advantages when compared with other methods. They can measure tens of thousands of different transcripts in the same reaction, they are semi-quantitative and sensitive to low-abundance transcripts if those are represented in a given array. 

There are several web resources that facilitate microarray data analysis (e.g., http://babelomics.bioinfo.cipf.es/) [34] or even web pages where the breeder can download experiments already performed and analyzed. There are also software packages specializated in microarrays analysis as the Bioconductor (http://www.bioconductor.org/help/work-flows/oligo-arrays/) or MeV (ttp://www.tm4.org/mev/) [35]. Probably one of the most useful database is Genevestigator (https://www.genevestigator.com/gv/ doc/plant_biotech.jsp) [36], which contains microarray data from different species. The most extensive data are from the model species *A. thaliana *[37], but an increasing number of studies in crops like maize, wheat, rice, barley, or soybean are already available. All published expression data are public and disposables in databases as GEO (http://www.ncbi.nlm.nih.gov/geo/) [38], ArrayExpress (http://www.ebi.ac.uk/array-express/) [39] or species specific databases. These data can be really useful when analyzing gene expression in these species or other crops [40]. 

Microarrays make use of the existing EST collections and genome sequence data. The vast increase provided by NGS in the number of sequences opens the possibilities of expression studies in a large number of species lacking previous sequence information. Also, deep NGS sequencing of RNA transcripts (RNA-seq) is emerging as an alternative to microarray studies to quantify gene expression [41, 42]. RNA-seq does not depend on genome annotation or on the probes contained in the array platform. This technology is also very useful to improve genome annotation, improving the detection of rare transcripts and splicing variants and the mapping of exon/intron boundaries. Moreover, RNA-seq avoids bias introduced during hybridization of microarrays and saturation level problems, haa a greater sensibility, and shows high reproducibility [41, 43]. This approach has been already used in different crops with different breeding objectives, leading to the identification of genes involved in several metabolic pathways, disease response, fruit development, etc. [44-47]. All these studies show the potential of RNA-seq for complex traits breeding. However, RNA-seq is an emerging technology and the methods used to analyze this kind of data are still being developed. 

### Mutant and Germplasm Collections in the Genomics Era: TILLING and EcoTILLING

Plant breeding requires genetic variability to be selected in order to increase the frequencies of favourable alleles and genetic combinations. Sources of natural genetic variability can be found within the crop, mostly in the form of landraces, and also in the wild relatives. Although many landraces have been substituted by modern and uniform cultivars and genetic erosion has taken place in wild materials, gene banks preserve many of these materials, which constitute an important reservoir of genetic variation useful for breeding [48]. 

Another important source of variability corresponds to the artificial mutant collections developed in several crop species. These artificial collections are created by radiation, chemical mutagenesis, or transgenic and insertion methods. Artificial mutations, mostly obtained by radiation and chemical methods, were used in plant breeding in the pre-genomics era, but new technologies are allowing the development of other types of collections [49]. For instance, the transferred DNA tagged lines and transposon tagged lines have been used to develop mutant collections in several species such as the model plant *Arabidopsis* (The Arabidopsis Information Resource; http://www.arabidopsis.org) or rice (International Rice Functional Consortium; http://irfgc.irri.org). Gene silencing technologies, using RNA interference, have also been used to create gene specific mutant collections in *Arabidopsis*, like the AGRIKOLA project (http://www.agrikola.org). The artificial mutant collections frequently contain variability not present in the natural collections, and so are also very useful for the study and development of new traits. 

In order to facilitate the identification of the accessions of interest in these collections, a genetic reverse approach has been used. Targeting Induced Local Lesions in Genomes (TILLING) [50] is able to identify all allelic variants of a DNA region present in an artificial mutant collection. A similar procedure called ecotype TILLING (EcoTILLING) [51] can be used to identify allelic variants for targeting genes in natural collections. These two methods are based on the use of endonucleases, such as CEL I or Endo I, that recognize and cut mismatches in the double helix of DNA [52, 53]. Since the TILLING and EcoTILLING techniques identify all allelic variants for a certain genomic region, the phenotypic characterization effort can be concentrated in a reduced number of accessions with different variants. Obviously, the success of the identification of variation useful for breeding programmes will depend on the right selection of target genes. The availability of sequences coming from NGS sequencing projects and the information provided by gene expression studies is significantly increasing the number and quality of candidates for TILLING and EcoTILLING studies.

These procedures have been successfully used in many crops [54]. For example, TILLING has been applied to *Arabidopsis *[55], *Lotus *[56], barley [57], maize [58], pea [59], and melon [60]. Rice was the first crop for which EcoTILLING was applied [61]. Subsequently, EcoTILLING has been used in other crops and wild relatives, like barley [62], wheat [63], or the wild peanut *Arachis duranensis* [64], using both genebank collections and natural populations [65]. These assays used gene targets involved in different processes. Many studies have been focused on detecting allelic variants in genes most related to organoleptic quality [66, 67] or disease resistance [68, 69]. 

### Re-Sequencing for SNPs Discovery and Use in Genotyping Platforms

One of the most interesting applications of NGS for plant breeders is the discovery of genetic variation. Now it is possible to sequence rapidly multiple individuals within a species with limited technical expertise and at minimal cost. The parallel development of computational pipeline tools is greatly accelerating the accurate mining of these sequences for genetic variants that can be converted into genetic markers, mainly microsatellites or simple sequence repeats (SSRs) and SNPs [70]. SSRs and SNPs are now the predominant markers in plant genetic analysis. SNPs are more abundant, stable, amenable to automation, and increasingly cost-effective, thus are fast becoming the marker system of choice in modern genomics research [71].

The genome-wide SNPs discovery by massive re-sequencing has been performed in model species with small genomes, such as *Arabidopsis thaliana*, where the 1001 Genomes project (http://www.1001genomes.org) [72] is attempting to unveil the whole-genome sequence variation in this reference plant. Similar re-sequencing efforts are becoming possible in rice, maize, grape, soybean, poplar etc. by sequencing sets of related genotypes, individually or pooled, within each species (elite cultivars, breeding lines, ecotypes, landraces, and/or weedy and wild relatives of a crop) [73-76]. The higher complexity in architecture and repeat content of these genomes has made necessary the use of approaches for genomic complexity reduction that also reduce sequencing cost [77]. Identification of SNPs is also very challenging in species with high levels of heterozygosity and/or with complex ploidy levels, as pseudo-SNPs are identified by misassembly of paralogs [78-80].

Both Roche 454 and Illumina GA have been mostly used for genome re-sequencing. The aligment difficulties often associtated to the use of short Illumina GA reads (Table **1**) are less problematic in species for which available reference genomes facilitates SNPs calling and genome positioning of genetic variation [81]. For most of these species, limited collections of SSRs and SNPs were available from early re-sequencing efforts, previous to the advent of NGS, but new genome-wide re-sequencing is enlarging the SNP pools and making them more representative of the range of natural variation.

For an increasing number of species with high societal or economic value NGS genome re-sequencing is providing the first SSRs and SNPs resources. Examples are the grain amaranths (*Amaranthus* sp.), important pseudocereals, appreciated for their nutritional quality and tolerance to abiotic stress [82], for which no prior genome information existed. In these cases the combination of several sequencing techniques, and the use of paired-end sequencing facilitates SNP discovery. Roche 454 and Illumina GA were combined for high-throughput SNP discovery in common bean [83] and also Solid was used to sequence diploid wheat species, which are donors of the subgenomes of modern hexaploid bread wheat [84]. 

Most of the effort in species lacking genomic resources is being made through transcriptome re-sequencing, as an alternative way of genome complexity reduction. Targeted amplicon re-sequencing is another strategy for discovering SNPs in specific genes [78]. 

One of the first examples of deep transcriptome sequencing was a study with two maize inbred lines [85]. This first study was followed by a large and rapidly increasing number of projects using non-model crops, some of them with large, complex, polyploid, and uncharacterized genomes, including forest trees, like *Eucalyptus* [86], oak [87], several polyploid crops, like oilseed rape [79], oats [80], coffee [88], and sweet potato [89], non-model grain legumes as chickpea and chickling pea [90], tomato [91], or several cucurbits, including *Cucurbita* spp. [21], cucumber [92], and melon [93]. 

These studies employ normalized/non-normalized cDNA libraries generated from single or multi-tissues samples, and derived from single or pooled genotypes, combined or not with multiplex identifier barcodes that allow allele origin identification. Sequencing is often focused on selected genotypes subjected to specific conditions, to detect SNPs in candidate genes involved in complex biological processes of interest to plant breeders. For example, the transcriptomes of two resistant and one susceptible lines of water yam, a major staple crop in Africa, under anthracnose infection, were successfully sequenced detecting SNPs in genes putatively involved in pathogen response [94]. Also, two alfalfa genotypes contrasting for cellulose and lignin content were sequenced, which allowed selecting SNPs useful to improve alfalfa as a forage crop and cellulosic feedstock [95]. 

The use of genome and transcriptome sequencing for SNP discovery has resulted in large SNPs collections in most of the crops. These large collections are being validated and applied for different purposes such as map construction, map saturation, genome-wide diversity studies, association mapping etc. (Table **3**). Some of the most important achievements will be described in later sections. 

There are many SNPs genotyping techniques, which are more or less appropriate for different scales of individuals/SNPs to be genotyped [107]. The implementation of marker-assisted breeding strategies often requires the generation of thousands of genotypes per population. Thus, one practical way of optimizing the use of these large SNPs collections is using them with cost-effective platforms for medium to high density genotyping. A large number of commercial platforms are available for semiautomated or fully automated SNP genotyping [108, 109]. Genotyping assays usually require a previous process of selection of a set of SNPs, among the hundreds/tens of thousands detected, that are appropriate for the assay objectives.

The Illumina GoldenGate assays have been the most widely used for mid-throughput applications. SNPs platforms with 384, 768, or 1536 SNPs are available for a number of species (Table **3**). Popularity is also increasing for the Sequenom Mass array and the KASPar genotyping chemistry [82, 110]. Expanded arrays with tens of thousands SNPs for high-throughput applications have been also developed with the Infinium technology in maize, grape, tomato, pine, and poplar and are under development in soybean and several *Rosaceae* crops (apple, peach, and cherry) [74, 111].

### Construction of High Density Genetic Maps 

One of the main applications of genomic advances is the development of high density genetic maps. The high-density map construction involves the location of hundreds or even thousand markers in the different linkage groups. In these maps the coverage should be very high and no large gaps must be present. NGS technologies and high-throughput genotyping platforms have allowed the improvement of genetic maps by increasing markers density. Several works include the integration of new markers, basically SNPs derived from re-sequencing studies, into previously developed genetic maps, both in diploid and polyploidy species [80, 112]. Golden Gate has been the most widely used platform. It has been estimated that this genotyping platform is 100-fold faster than gel-based methods for increasing 2-3 times maize map density [101]. Also Sequenom-based SNP-typing assays are starting to be applied. In a recent study, a total of 1.016 SNPs, identified *via* comparative next-generation transcriptomic sequencing, were successfully mapped by genotyping 297 maize recombinant inbred lines (RILs) [113]. Other genotyping strategies based on arrays hybridization, such as the single-feature polymorphisms (SFP), variants detected by a single probe in oligonucleotide arrays, are speeding up genetic map construction. This technique has been used for the construction of a high-density linkage map in species poorly characterized, like *Eucalyptus* [114]. The newly developed maps, enriched in sequence-based markers are facilitating comparative mapping. Recent examples are high density SNPs maps of barley compared with wheat and rice [98, 115]. 

The decrease of sequencing costs is also allowing the detection of new types of genetic markers useful for increasing the density of genetic maps.In this respect, restriction-site associated DNA (RAD) is a kind of marker which detects genetic variation adjacent to restriction enzyme cleavage sites across a target genome. These markers are produced after NGS sequencing of genomic libraries obtained after digestion with different restrictases. As an example of the utility of this technique, a total of 445 RAD markers distributed across all seven barley chromosomes were located, which was very useful for linkage map construction in this crop [116].

The markers derived from NGS can also be useful to position sequence scaffolds onto physical maps. In this respect, a new method combining deep sequencing and the bin mapping strategy has been described [117]. The SNPs identified by re-sequencing genomic libraries from selected progeny individuals, derived from a cross between two closely related diploid strawberry species, were used to anchor 92.8% of the *Fragaria *genome to the genetic map. Results highlighted the potential of this methodology to obtain a robust framework for the anchoring of the genome sequence without the requirement of a high density physical mapping or a well-saturated genetic map. 

Whole-genome re-sequencing at different coverage levels is being increasingly applied for map construction using different strategies. As an example, a genetic map for rice has been constructed using whole genome re-sequencing of 150 RILs [118]. These authors concluded that the sequencing-based method was approximately 35 times more precise in recombination breakpoint determination than PCR-based markers maps. Also, the whole genome of 128 chromosome segment substitution lines (CSSLs) of rice was re-sequenced and used it for the construction of an ultra-high quality physical map in this crop [119]. Based on low coverage re-sequencing, a new mapping strategy that allows inferring the parental genotypes of the assayed RILs population has been proposed [120]. An ultra-high density linkage map was obtained with this method and the quality of the map was evaluated by using it to identify a QTL controlling grain width. Further applications of new sequence-based denser genetic maps to QTL discovery and marker assisted selection (MAS) will be discussed later.

## TOWARDS A GENOMICS-BASED PLANT BREEDING 

### Genome-Wide Genetic Diversity Studies 

One of the main challenges in agricultural genetics is to access and use the tremendous genetic variation present in germplasm collections and in the wild relatives. A significant part of this variation remains untapped because of the difficulties in effectively identifying genetic differences in large collections. Some traits, with high heritability and of simple characterization, are easy to select for. However, desirable allelic variants and genetic combinations for complex traits are difficult to identify. Recent advances in genotyping are enabling genome-wide diversity studies capable of better capturing the spectrum of variability in natural and breeding populations. 

Most of the mid- to high-throughput genotyping platforms described above are being used for studies on diversity and population structure in the corresponding crops (Table **3**). By using representative diversity panels, polymorphism rates for individual SNP markers, minor allele frequencies (MAFs), etc. are estimated, facilitating the selection of those SNPs with biological interest and highly polymorphic in the different groups. For example, the Infinium arrays developed in some of these crops are being used to create haplotype maps for vast germplasm collections, such as the 18,000 accessions of the USDA soybean germplasm collection [121]. 

Haplotype maps (hapmap) of entire collections are useful to identify rare, potentially valuable, alleles. Hapmap projects are undergoing in a number of species such as the “*rice diversity project*” (http://www.ricehapmap.org/index.aspx) aimed to develop a 10,000 SNP chip for rice and create a haplotype map to document the differences in allelic variation within and between the different subpopulations of *O. sativa* and its progenitor *O. rufipogon*. Large-scale genetic diversity studies have also been accomplished in maize. Gore *et al*. [122] identified and genotyped several million sequence polymorphisms among 27 diverse maize inbred lines. This study allowed the discovery of regions with highly suppressed recombination that appear to have influenced the effectiveness of selection during maize inbred development and may be a major component of heterosis. Also, highly differentiated regions were found that probably contained *loci* that are key to geographic adaptation. Also in legumes, the Medicago HapMap Project, that consist in the sequencing the whole-genomes of 30 inbred lines, will explore the genetic basis of symbiosis, creating a robust platform for genome-scale association mapping [123].

The diversity panels can include representatives of close or more distantly related species to check if these sequence-based SNP assays also work in related species [74, 82]. Sometimes sets of SNPs specifically developed for detecting genetic diversity among closely related cultivars are used in genotyping platforms. For example, despite the large amounts of SNPs available in rice obtained from the comparison of the two reference genomic sequences (one *japonica* and one *indica* variety) [124], extremely low levels of DNA polymorphism were detected within *japonica* cultivars. A whole-genome sequencing of an elite Japanese rice cultivar, closely related to the reference *japonica* variety, has been conducted and the SNP information obtained by comparison of the two *japonica* sequences was applied to develop a high-throughput genotyping array used for genotyping a set of representative Japanese cultivars [125]. These experiments are useful for understanding the role of selection and breeding in the distribution of genetic variation across the crop genome. In fact, this assay led to the identification of several haplotype blocks which are inherited from traditional landraces to current improved varieties. Moreover, it was found that, as predicted, modern breeding practices have generally decreased genetic diversity. On the practical level, the distribution of genetic diversity in modern cultivars plays an important role in the choice of specific mapping and crop improvement strategies. 

Genome-wide survey of genetic diversity is useful to elucidate the causative genetic differences that give rise to observed phenotypic variation providing a foundation for dissecting complex traits through genome-wide association studies. However, its utility is limited if phenotypic data are not available. Not just genomics and transcriptomics, but the other 'omics' disciplines, like proteomics and metabolomics, are useful to understand how the changes in the genotype lead to differences in the final phenotype. Phenomics, which uses high-throughput technologies to characterize germplasm, is being developed and will help to deal with this issue [126].

### Identification of Molecular Markers Linked to Single Genes and QTLs

NGS and high-resolution maps have led to a significant improvement in the identification of molecular markers linked to specific genes and to QTLs. The most important advantage comes from the dense genome coverage, which allows the identification of markers closely linked to any target genomic region, with the advantages that this tight linkage provides. 

Methods already used in the pre-genomics era to facilitate the identification of markers linked to single loci, such as bulked segregant analysis (BSA), are now optimized. For example, a GoldenGate assay has been combined with BSA to significantly accelerate mapping of the dominant resistance *locus* to soybean rust *Rpp3 *[127]. In this respect, there is an increasing number of reports on exploitation of NGS technologies to identify molecular markers tightly linked to major genes. For example, a fine genetic mapping of the single dominant scab resistance gene (*Ccu*) in RILs of cucumber (*Cucumis sativus*) has been conducted [128]. The resistant cucumber genome was sequenced with Solexa/Illumina NGS and compared with the susceptible cucumber genome. As a result, three insertion/deletion (indel) markers closely linked to the *Ccu* *locus* where obtained. A detailed study of the region delimited by markers revealed four resistance gene analogs as possible candidates for *Ccu*. 

QTL detection has traditionally been conducted by linkage mapping. NGS technologies are significantly contributing to increase accuracy in detection of QTLs. They allow increases in many orders of magnitude of the number of markers mapped, ensuring high mapping resolution, and also aid in the development of mapping populations, such as RILs, near isogenic lines (NILs), and CSSLs, more appropriated for QTLs detection. These populations have conventionally been constructed and genotyped using a limited number of markers. 

There are increasing reports describing accurate QTLs mapping with different NGS or high-throughput genotyping strategies. For example, a high density rice map constructed by whole-genome re-sequencing of a RILs population, was used to identify four QTLs controlling plant height [90]. On a different study [129] an ultra-high density genetic map based on SNPs, obtained with Illumina GA, was compared with a linkage map based on RFLPs/SSRs in rice. The positions of several cloned genes, two major QTLs for grain length and grain width, and a QTL for pigmentation were evaluated in a RIL population, arising the expected result that the SNPs map detected more QTLs and more accurately than a RFLPs/SSRs based linkage map. 

QTL detection based on the linkage analysis method has the disadvantage that the number of recombination events is limited to the generations needed to develop the mapping population. Association mapping or linkage disequilibrium (LD) mapping is a new powerful approach to map complex traits. This method identifies genetic *loci* associated with phenotypic trait variation in a collection of individuals. Association mapping uses the natural diversity, which represents many more recombination events occurred in the history of the population, providing better resolution. Nowadays, two association mapping methodologies are in use: candidate gene association, where a good understanding of the biochemistry and genetics of the trait is needed, and whole genome scan, also called genome-wide association (GWA) studies. New genomic advances are providing the higher density of genetic markers required to ensure enough coverage to detect linkage between markers and a causal *locus*. Also the decrease of sequencing costs (Table **1**) has allowed the use of whole genome sequencing in current studies [130].

Nevertheless, association mapping is just rising in model species and major crops. Maize is the most widely studied crop regarding association analysis. Many candidate genes have been successfully associated to morphological or quality traits. As an example, candidate genes *Dwarf8*, *Vgt1* and *ZmRap2.7* were successfully associated to flowering time [131]. Other candidate genes have been associated, among others, to forage quality, carotenoid content, oil content and kernel quality [132-135]. GWA studies have been more limited, probably due to the large genome of maize (2300 Mbp) and the great number of markers needed to cover it. The first study identified a fatty acid desaturase gene (*fad2*) associated with increased oleic acid levels [99]. More recently, other authors found 32 QTLs associated with southern leaf blight disease resistance [100].

Examples of association mapping approaches in other crops are more limited. Studies based on the candidate gene approach have been reported in some crops, like grape, or conifers [102, 106]. However, GWA studies have only been developed either in the model species *A. thaliana *[136] or in major crops such as rice [96], barley [97], or wheat [104]. Some articles also describe successful mapping processes combining classical linkage mapping and association mapping [137]. Although genetic association mapping is in its early steps, it is a promising tool for the dissection of complex traits in crop plants.

### Marker Assisted Selection 

#### Marker Assisted Backcross Selection

Marker assisted selection (MAS) is an indirect process where selection is carried out on the basis of a marker instead of the trait itself. The successful application of MAS relies on the tight association between the marker and the major gene or QTL responsible for the trait. As we have described before, the new genomic tools accelerate the identification of markers tightly linked to target genomic regions. On the other hand, the new dense genotyping platforms available today accelerate the genotyping of large amounts of samples during the MAS process in a rapid and economically feasible manner. MAS can take benefit from these technologies, speeding up the release of new varieties.

In spite of the close linkage between the marker and the gene, the possibility of recombination limits the use of MAS. The use of intragenic markers, also called functional markers, can help to overcome this limitation [138]. NGS sequencing projects produce large collections of functional markers. These markers enhance real gene assisted breeding, reducing the possibility of losing the desirable trait due to recombination. This is today feasible in many crop species in which NGS cDNA sequencing is being conducted. Some of these studies perform expression profiling, identifying candidates and associated gene targeted markers. 

MAS is also frequently applied to perform background selection in the context of backcrossing programmes. Background selection consists in the identification of plants with lower contents in donor genome to continue the breeding scheme, in order to achieve the recovery of the recipient genome. The use of background markers facilitates the quick recovery of the recurrent parent genome [139]. Background selection is being used extensively in rice breeding. High-density genome maps are being effectively used in background analysis. For example, background selection integrated with foreground selection of bacterial blight resistance (*xa13* and *Xa21* genes), amylose content (*waxy* gene) and fertility restorer gene has been performed in order to identify superior lines with maximum recovery of Basmati rice genome along with the quality traits and minimum non-targeted genomic introgressions of the donor chromosomes [140].

In some cases, the problem of recovering the genetic background of the recurrent parent arises because of the linkage drag, that is, the introgression of chromosome regions with deleterious effects which are tightly linked to the gene or QTL of interest. The detection of QTLs responsible of the negative effects and the localization of molecular markers tightly associated to them can be an efficient way to break the genetic drag. A well known example concerns canola (rapeseed) breeding, which began with the discovery of germplasm with low erucic acid content in seeds of a spring forage cultivar in the 1950’s. The problem arose because a high association between low erucic acid content and low seed oil content exists. The recent availability of high-density molecular maps has allowed the detection of several QTLs associated to both traits. Moreover, the identification of molecular markers very tightly linked to the QTLs made possible to break the linkage drag between the low oil content and erucic acid concentration in seeds in the process for breeding new high seed oil content canola cultivars [141]. 

Frequently, current breeding programmes involve the introgression of more than one gene or QTL controlling traits of interest into one genetic background, in a process that is called pyramiding. The most useful application of MAS in the process of pyramiding is related to the introgression of different genes or QTLs whose effect on the phenotype is undistinguishable. The accumulation of genes from different sources which confer resistance against the same disease is an example, and is indeed one of the most widespread applications of gene pyramiding [142]. The main advantages of recent advances in plant genomics incorporated into gene pyramiding will be related to two different aspects. On one hand, the number of plants to be analyzed in a gene pyramiding programme must be increased as the number of *loci* of interest is higher, to ensure with a reasonable likelihood that the genotype combining favorable alleles is present in the population [143]. In this sense, the availability of genotyping platforms will provide the possibility to screen larger generations. On the other hand, the efficiency of the process strongly depends on the tightness of the linkage between markers used and the target genes or QTLs. Again, identification of functional markers will circumvent this limitation. 

#### ‘Breeding by Design’

The possibility to predict the outcome of a set of crosses on the basis of molecular markers information is known as ‘breeding by design’ [6]. The process includes three steps: mapping *loci* involved in all agronomically relevant traits, assessment of the allelic variation at those *loci*, and, finally, breeding by design. In the method as initially described by Peleman and van der Voort [6], the first step was proposed to be completed by either using mapping populations segregating for the trait of interest or based on a candidate gene approach (mainly exploiting information from model plant species and increasing understanding of gene function). Also linkage disequilibrium (LD) mapping was suggested, focused on the region previously identified as related to the trait (‘targeted LD mapping’). Currently, as previously discussed, other possibilities such as GWA studies allow a more efficient way to accomplish this first step, avoiding limitations of biparental populations. The second step of the process consists in the identification of allelic variation for the *locus *of interest and the assignation of the phenotypic value to each of them. This step cannot be based on biparental populations, given that only two alleles per *locus *are segregating in this case. The analysis should then include plant materials representing the variability of the species. Genotypic and phenotypic data for each plant are required. 

As previously stated, high level of saturation with markers is not the limiting factor in most cases, and so currently the restrictions mostly come from the phenotyping step. Strictly speaking, ‘breeding by design’ exploits information obtained in the previous steps: once the *loci* of interest have been mapped, and the contribution of each allelic variant has been determined, crosses can be established to generate superior genotypes which combine all favourable alleles. Application of this breeding strategy has been used for different crops and with different objectives, such as breeding for heading date in rice [144] or seed length in soybean [145]. This procedure has also been used in patent applications; as an example, ‘breeding by design’ has been reported as part of the development of higher quality maize varieties. However, the most effective application of the ‘breeding by design’ approach will come from the incorporation of the most advanced genomic tools into the process, which will allow the improvement of the predictions. 

#### Genomic Selection

**Fig. (1):**
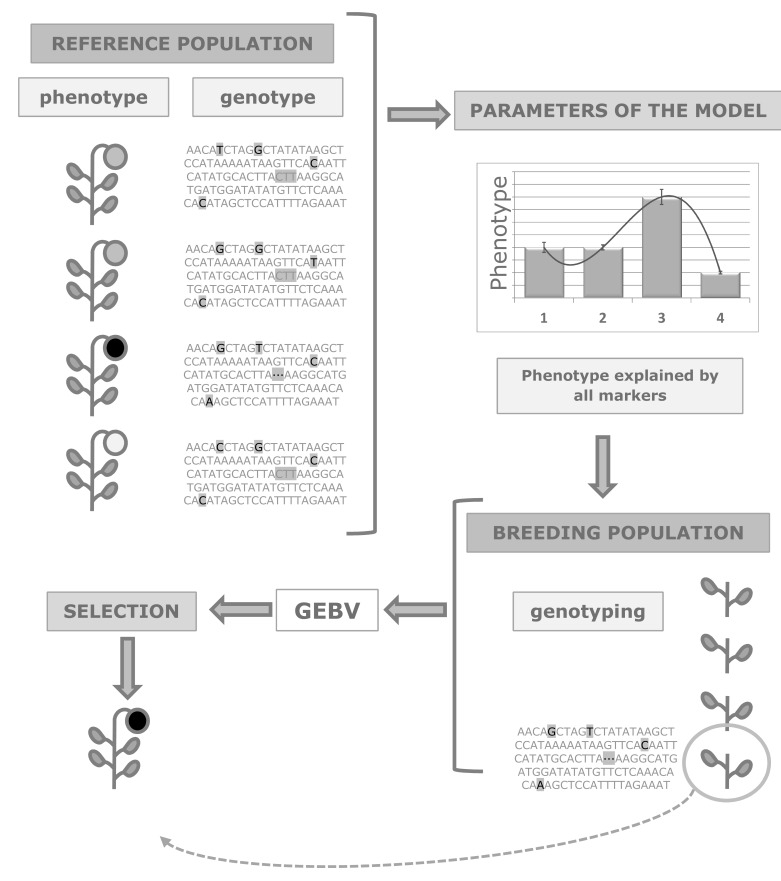
Genomic selection scheme. Information on phenotype and genotype for a reference population allows estimating parameters for the model. This model explains phenotype based on all markers analyzed. The model predicts the phenotype of plants in a breeding population on the basis of the genotyping results: this is the genomic estimated breeding value (GEBV), used to select the desired phenotypes.

MAS strategies described so far require the identification of markers associated to the traits of interest. This represents one of the weaknesses of traditional MAS approaches [146]. Nevertheless, MAS can also be applied eluding this step, using an approach known as genomic (or genome-wide) selection (Fig. **1**). The method was first described in 2001 [147], as an attempt to exploit information generated from emerging genotyping technologies. Genomic selection is based on simultaneous estimation of effects on phenotype of all *loci*, haplotypes, and markers available. The difference with other MAS methods relies on the fact that no previous selection of markers with effects on phenotype is developed [148]. Genomic selection requires the availability of phenotypic and genotypic data for the reference population. This data set will allow estimating the parameters for the model, so that the differences at the phenotype level are explained by the markers analysed. Once the model is established, application to breeding populations makes possible to determine the genomic value of each individual, i.e., the expected phenotype based on the genotypic data. The requirement is the availability of enough molecular markers to provide good genome coverage [5, 146].

Simulation studies carried out using maize proved the usefulness of genomic selection applied to an initial cross between an adapted line and exotic germplasm. With 512 markers and a reference population of 288 F_2_ plants evaluated in six different environments, it was possible to obtain good selection response after 7-8 generations. [149]. Also with maize, simulations showed that response to selection was 18 to 43% larger for genomic selection than for marker assisted recurrent selection [150]. Response obtained when using genomic selection can be lower than response by phenotypic selection. However, the reduction in cycle length due to early MAS results in an increase of gain per time unit. This reduction is even more accused for species with a long generation interval, such as tree species [148].

The availability of phenotypic databases for different crops has allowed the comparison of predictions about the genotypic value obtained using genomic selection with the true genotypic value as shown by the phenotypic manifestation of the trait. In a study developed with phenotypic and genotypic data from *Arabidopsis*, maize and barley, results obtained were more accurate when genome-wide selection was carried out, if compared with results derived of previous selection of markers with effects on the phenotype [151]. 

Although when applying genomic selection there is no need to previously identify QTLs controlling a certain trait, the utilization of this approach allows detecting the chromosome regions involved in a given trait, as markers with greater effect on the phenotype will indicate the presence of a QTL for this trait [152]. Some studies go one step farther and propose the application of MAS prior to phenotyping. This approach involves the use of *prior indices*, i.e., marker selection indices which have been constructed from a given phenotyped and genotyped population and are applied to different populations which have not been phenotyped [129]. The decrease in the costs of genotyping provides the appropriate scenario for this strategy to become cost-effective.

In any case, even the identification of the QTLs responsible for a certain trait does not imply the identification of the gene or genes controlling the trait itself or the understanding of the mode of action. Models applied in genomic selection are useful to predict breeding values and, in some cases, detect regions associated to a trait, but further work is necessary from this point to identify the gene or genes responsible for the phenotypic variability observed. From plant breeders’ perspective, the availability of molecular markers which allow MAS to be applied is generally sufficient. However, development of the new high throughput -omics technologies has provided breeders with new strategies to search for candidate genes, mainly based on microarray for differential gene expression, being the possibility to explore more genes the most important advantage. Future exploitation of these strategies could facilitate the identification of candidate genes underlying the traits of interest and make MAS more efficient.

## CONCLUSIONS

For some major crops the pace experimented for genetic gains in yield and other complex traits in the 20^th^ century will be difficult to be maintained if only existing pre-genomics technologies are used [153]. However, plant breeding is a dynamic science and, fortunately, genomics resources and tools are already available and are helping to give another quantitative leap in plant breeding. In this respect many advances are already taking place, and the superdomestication, i.e., “the processes that lead to a domesticate with dramatically increased yield that could not be selected in natural environments from naturally occurring variation without recourse to new technologies” [10], will require the combination of conventional breeding with crop genomics. Also, genomic tools and approaches will help conventional breeding in achieving important advances in the breeding of crops that from the point of view of genetic improvement have remained either orphaned or neglected [8]. Therefore, while conventional pre-genomics plant breeding has been, is, and will be successful at improving our crops, the application of genomic tools and resources to practical plant breeding will push forward the genetic gains obtained by breeding programmes. New genomic advances, many of which are already being developed, will make easier for breeders to obtain new cultivars with improved characteristics, either by facilitating selection or by improving the variation available for breeders by using precision breeding approaches. In particular, the present and new genomics tools are of great value for the genetic dissection and breeding of complex traits.

## Figures and Tables

**Table 1.** Comparison of the Main Characteristics of the Conventional Sanger and Some of the Most Currently Used Next Generation Sequencing (NGS) Technologies and Approximate Sequencing Cost (in US $ Per Mbp)

Technology	Read Length (bp)	Mbp per run	Cost ($/Mbp)
Sanger	1000	0.001	3000.00
454 Roche	450	450	66.00
Illumina Hi-Seq2000	100	270000	0.07
Solid 5500xl	50	270000	0.07

**Table 2.** Some Important Databases and Repositories of Genomic Information of Interest for Breeders

Database	Description	URL
Genbank	General public sequence repository	http://www.ncbi.nlm.nih.gov/genbank/
EMBL	General public sequence repository	http://www.ebi.ac.uk/embl/
DDBJ	General public sequence repository	http://www.ddbj.nig.ac.jp
UniProt	Protein sequences and functional information	http://www.uniprot.org/
NCBI	Biomedical and genomical information	http://www.ncbi.nlm.nih.gov/
Gene Index Project	Transcriptome repository	http://compbio.dfci.harvard.edu/tgi/
GOLD	Repository of genomes databases	http://genomesonline.org/cgi-bin/GOLD/bin/gold.cgi
Phytozome	Genomic plant database	http://www.phytozome.net/
Plantgdb	Genomic plant database	http://www.plantgdb.org
CropNet	Genomic plant database	http://ukcrop.net/
SGN	Solanaceae information resource	http://solgenomics.net/
Gramene	Grass information resource	http://www.gramene.org/
MaizeGDB	Maize infornation resource	http://www.maizegdb.org/
Tair	Arabidopsis information resource	http://www.arabidopsis.org/
CotthonDB	Cotton information resource	http://cottondb.org/
CPGR	Phytopathogen genomic resource	http://cpgr.plantbiology.msu.edu/

**Table 3.** Some Examples of the Utility of Molecular Markers Developed by Means of High-throughput Genomics Techniques for the Breeding of Important Crops

Crop	Markers	Plant Material	Use for Breeding	Reference
Rice (*Oryza sativa*)	~3.6·10^6^ SNPs	517 rice landraces	Association studies for 14 agronomic traits	[96]
Barley (*Hordeum vulgare*)	1,536 SNPs	768 breeding lines	Association studies for *Fusarium* head blight resistance	[97]
	3,072 SNPs	336 DH lines and 213 germplasm selections	High-density genetic map construction and MAF estimation	[98]
Maize (*Zea mays*)	8,590 SNPs	553 elite maize inbred lines	Association studies for oleic acid content	[99]
	1,106 SNPs	5,000 RILs	Association studies for resistance to southern leaf blight	[100]
	1,536 SNPs	154 maize inbred lines	Diversity studies	[101]
Grapevine (*Vitis vinifera*)	94 SNPs and 7 indels	148 grape varieties	Association studies for muscat flavor candidate gene VvDXS	[102]
	9000 SNPs	10 cultivated *Vitis vinifera *and 7 wild *Vitis* spp.	Diversity and population structure studies	[74]
Pea (Pisum sativum)	384 SNPs	91 RIL mapping population and 373 Pisum accessions	Linkage map construction and diversity studies.	[103]
Wheat (*Triticum aestivum*)	874 DArT markers	winter wheat core collection of 96 accessions	Association studies for 20 agronomic traits	[104]
	1,536 SNPs	478 spring and winter wheat cultivars	Diversity studies	[105]
White spruce (*Picea glauca*)	944 SNPs	492 individuals	Association studies with 549candidate genes and 25 wood quality traits	[106]

## ABBREVIATIONS

AFLP: = Amplified Fragment Length Polymorphism
BED: = Browser Extensible Data
BSA: = Bulked Segregant Analysis
CSSL: = Chromosome Segment Substitution Line
DArT: = Diversity Arrays Technology
EcoTILLING: = Ecotype TILLING
EST: = Expressed Sequence Tag
GFF: = General Feature Format
GWA: = Genome Wide Association
LD: = Linkage Disequilibrium
MAF: = Minor Allele Frequency
MAS: = Marker Assisted Selection
MPSS: = Massively Parallel Signature Sequencing
NGS: = Next Generation Sequencing
NIL: = Near Isogenic Line
QTL: = Quantitative Trait Locus
RAD: = Restriction-Site Associated DNA
RIL: = Recombinant Inbred Line
RNA-seq: = Sequencing of RNA Transcripts
SAGE: = Serial Analysis of Gene Expression
SAM: = Sequence Alignment and Modelling
SPF: = Single-Feature Polymorphism
SNP: = Single Nucleotide Polymorphism
SSR: = Simple Sequence Repeat
TILLING: = Targeting Induced Local Lesions on Genomes
VCF: = Variant Call Format
